# Supplementary figures and images for: Noncovalent antibody catenation on a target surface greatly increases the antigen-binding avidity
Source: eLife. 2023 May 30;12:e81646. doi: 10.7554/eLife.81646 (PMC10229114; doi:10.7554/eLife.81646)

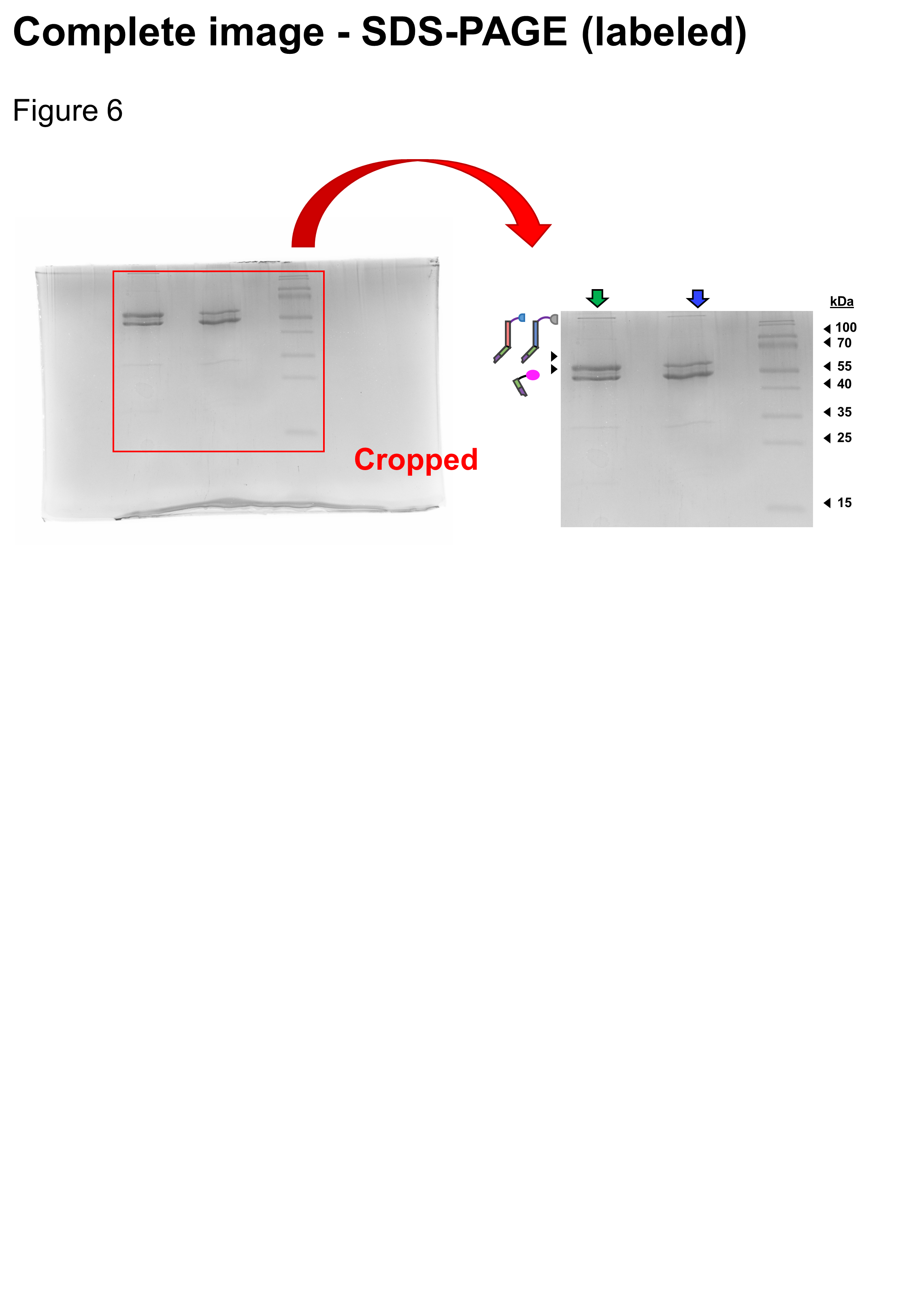

Supplement: Figure 6—source data 1. [file elife-81646-fig6-data1.zip › Fig6_SourceData_1_labeled.TIF]

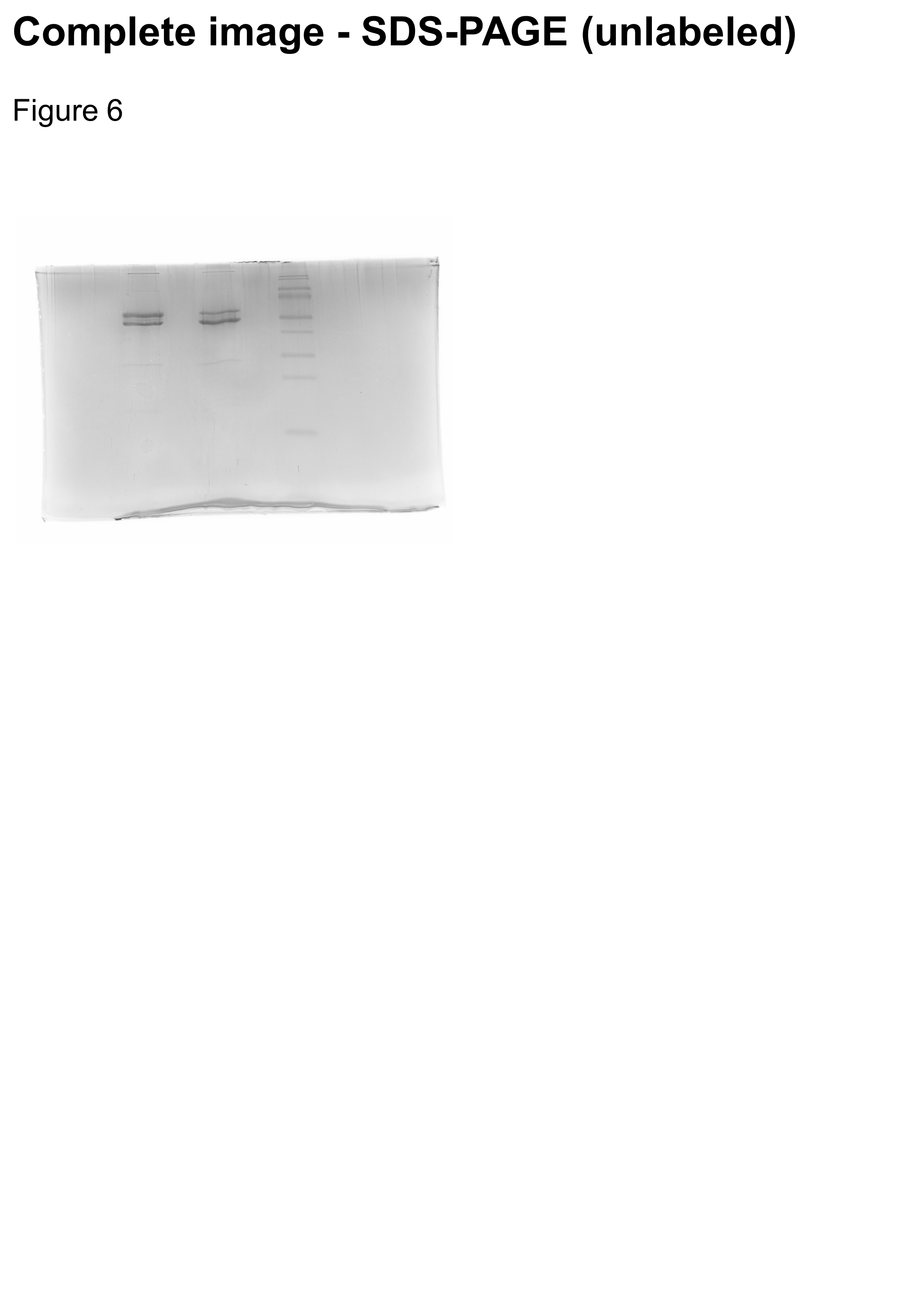

Supplement: Figure 6—source data 1. [file elife-81646-fig6-data1.zip › Fig6_SourceData_1_unlabeled.TIF]

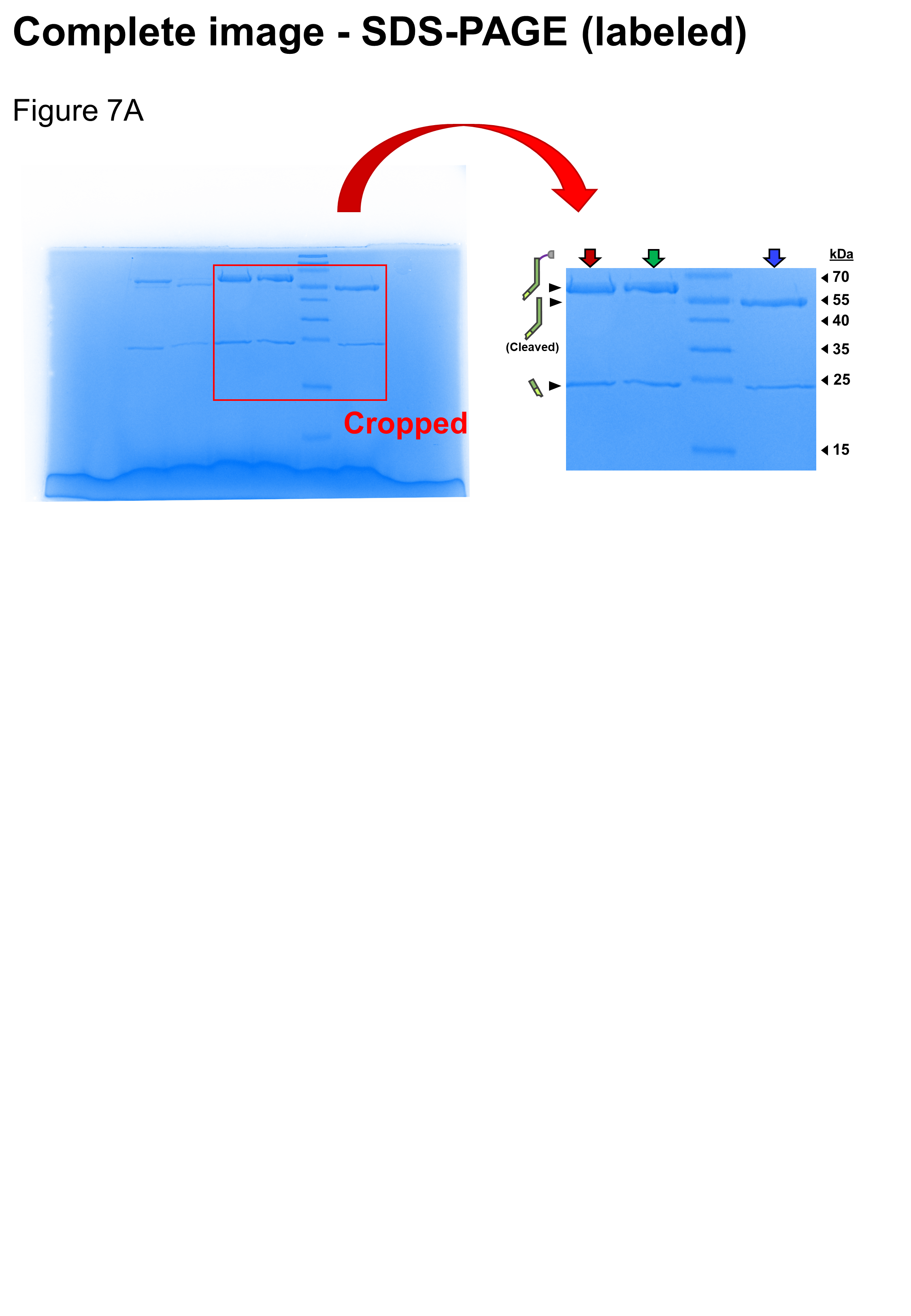

Supplement: Figure 6—figure supplement 1—source data 1. [file elife-81646-fig6-figsupp1-data1.zip › Fig6_FigureSupplement1_SourceData_1_labeled.TIF]

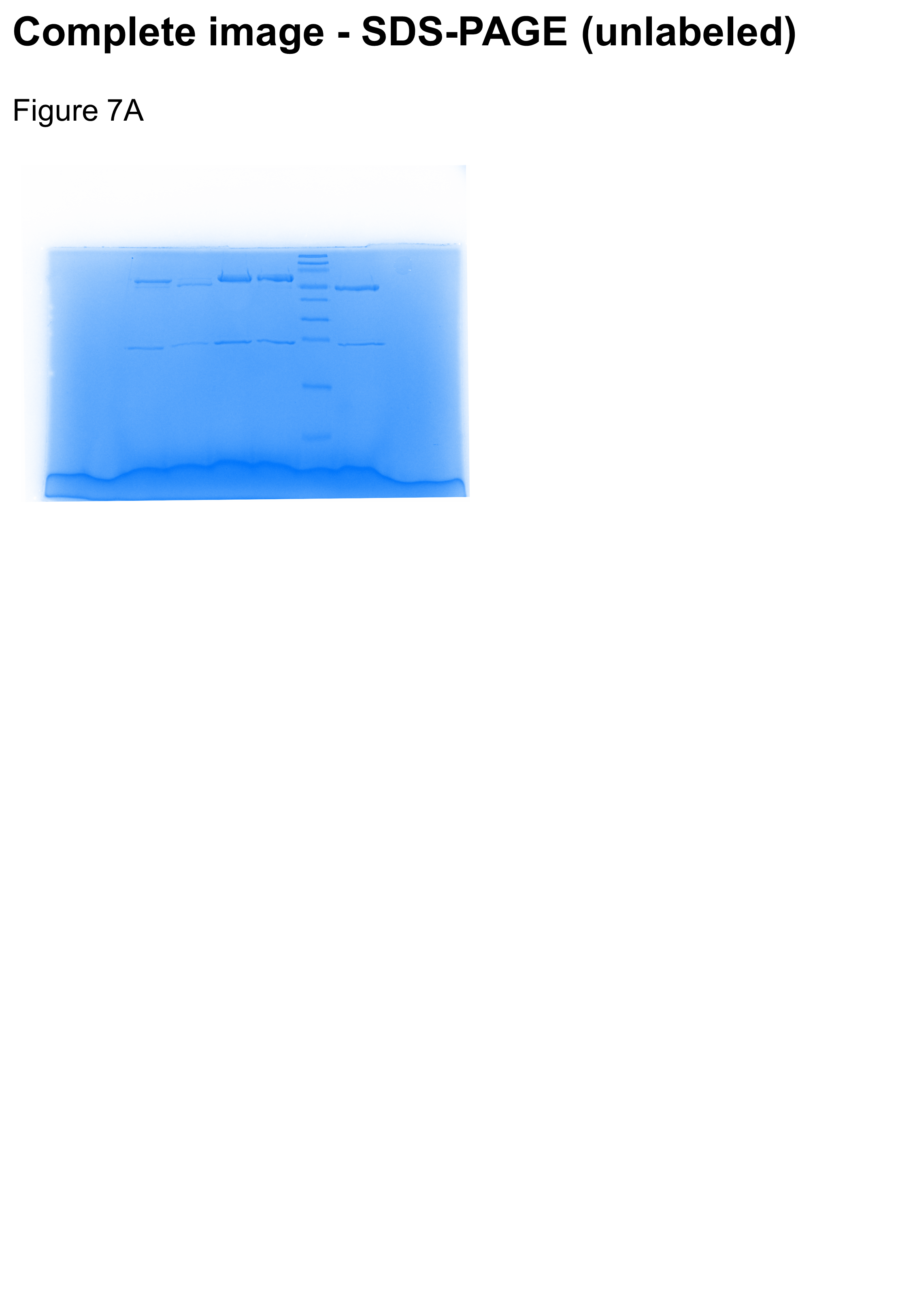

Supplement: Figure 6—figure supplement 1—source data 1. [file elife-81646-fig6-figsupp1-data1.zip › Fig6_FigureSupplement1_SourceData_1_unlabeled.TIF]

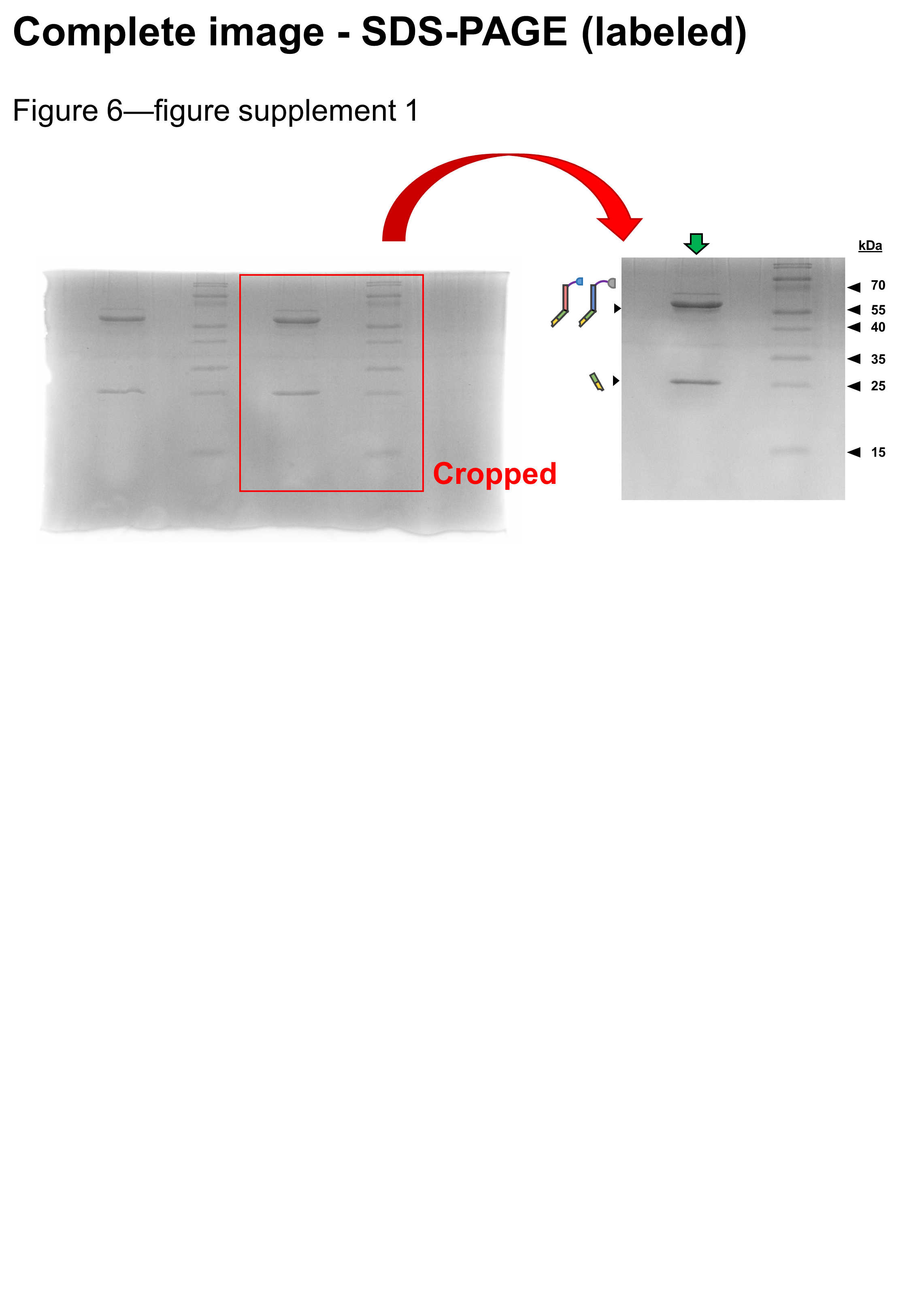

Supplement: Figure 7—source data 1. [file elife-81646-fig7-data1.zip › Fig7_SourceData_1_labeled.TIF]

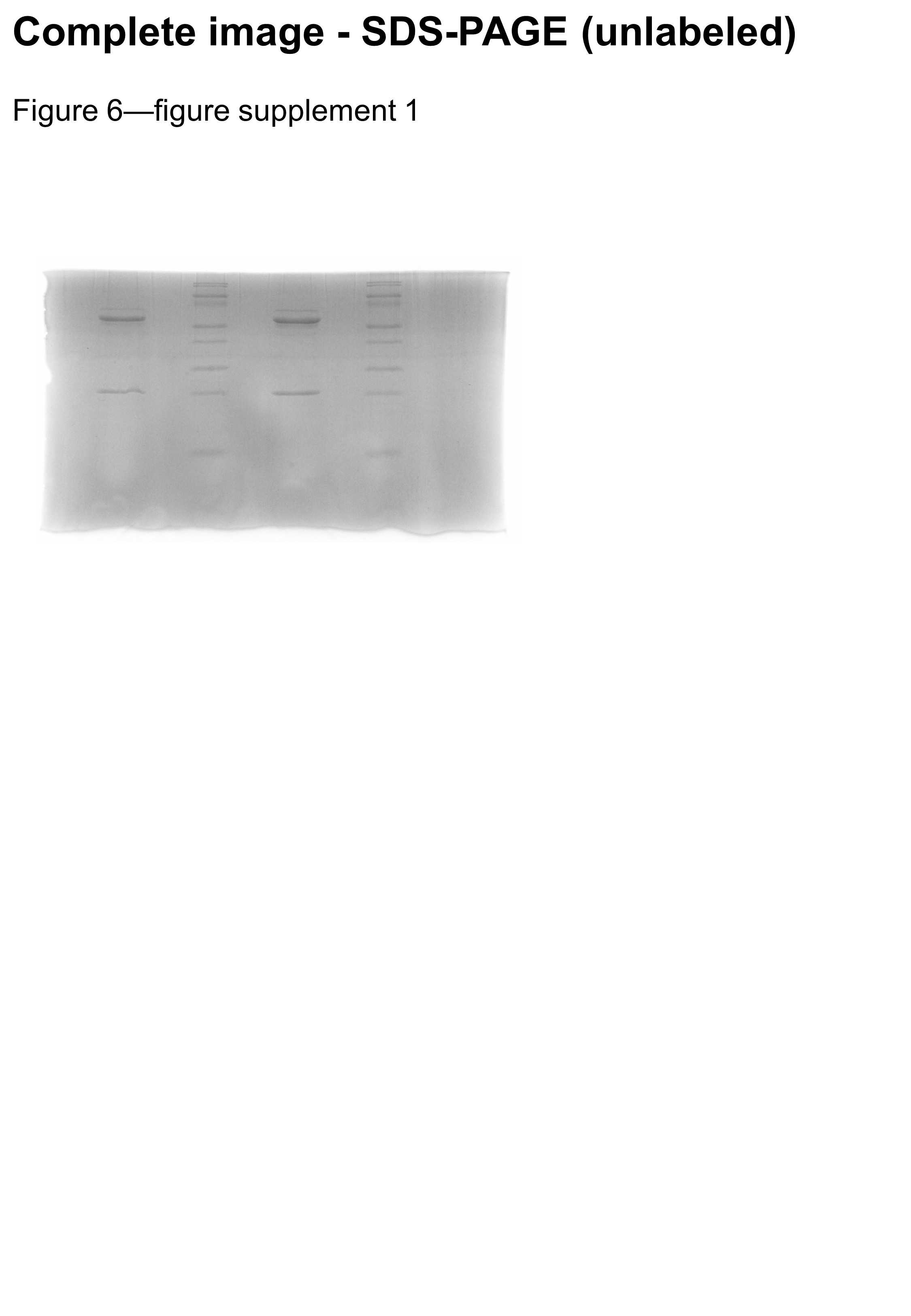

Supplement: Figure 7—source data 1. [file elife-81646-fig7-data1.zip › Fig7_SourceData_1_unlabeled.TIF]
